# Molecular diagnosis of patients affected by mucopolysaccharidosis: a multicenter study

**DOI:** 10.1007/s00431-019-03341-8

**Published:** 2019-02-26

**Authors:** Alessandra Zanetti, Francesca D’Avanzo, Laura Rigon, Angelica Rampazzo, Daniela Concolino, Rita Barone, Nicola Volpi, Lucia Santoro, Susanna Lualdi, Francesca Bertola, Maurizio Scarpa, Rosella Tomanin

**Affiliations:** 1 Laboratorio di Diagnosi e Terapia delle Malattie Lisosomiali, Department of Women’s and Children’s Health, University of Padova, Padova, Italy; 2 Fondazione Istituto di Ricerca Pediatrica Città della Speranza, Padova, Italy; 3 Department of Pediatrics, University of Catanzaro, Catanzaro, Italy; 4 Department of Clinical and Experimental Medicine, Child Neurology and Psychiatry, University of Catania, Catania, Italy; 5 Department of Life Sciences, University of Modena and Reggio Emilia, Modena, Italy; 6 Department of Clinical Sciences, Division of Pediatrics, Polytechnic University of Marche, Ospedali Riuniti, Presidio Salesi, Ancona, Italy; 7 Laboratorio di Genetica Medica e Biobanche, Istituto Giannina Gaslini, Genoa, Italy; 8 School of Medicine and Surgery, University of Milano Bicocca, Monza, Italy

**Keywords:** Lysosomal storage disorders, Mucopolysaccharidoses, Genetics analyses, Genotype-phenotype correlation, ACMG classification

## Abstract

Mucopolysaccharidoses (MPS) are a subgroup of 11 monogenic lysosomal storage disorders due to the deficit of activity of the lysosomal hydrolases deputed to the degradation of mucopolysaccharides. Although individually rare, all together they account for at least 1:25,000 live births. In this study, we present the genetic analysis of a population of 71 MPS patients enrolled in a multicenter Italian study. We re-annotated all variants, according to the latest recommendations, and re-classified them as suggested by the American College of Medical Genetics and Genomics. Variant distribution per type was mainly represented by missense mutations. Overall, 10 patients had received no molecular diagnosis, although 6 of them had undergone either HSCT or ERT, based on clinical and enzymatic evaluations. Moreover, nine novel variants are reported.

*Conclusions*: Our analysis underlines the need to complete the molecular diagnosis in patients previously diagnosed only on a biochemical basis, suggests a periodical re-annotation of the variants and solicits their deposition in public databases freely available to clinicians and researchers. We strongly recommend a molecular diagnosis based on the analysis of the “trio” instead of the sole proband. These recommendations will help to obtain a complete and correct diagnosis of mucopolysaccharidosis, rendering also possible genetic counseling.

**Table:** 

**What is known**	
*• MPS are a group of 11 metabolic genetic disorders due to deficits of enzymes involved in the mucopolysaccharides degradation.*	
*• Molecular analysis is commonly performed to confirm enzymatic assays.*	
**What is new**	
*• Eighty-six percent of the 71 patients we collected received a molecular diagnosis; among them, 9 novel variants were reported.*	
*• We stress the importance of molecular diagnosis in biochemically diagnosed patients, encourage a periodical re-annotation of variants according to the recent nomenclature and their publication in open databases.*

**Electronic supplementary material:**

The online version of this article (10.1007/s00431-019-03341-8) contains supplementary material, which is available to authorized users.

## Introduction

Mucopolysaccharidoses (MPS) are very rare, monogenic, metabolic disorders due to the deficit of the lysosomal enzymes normally degrading mucopolysaccharides or glycosaminoglycans (GAG), this causing their pathological accumulation in most tissues and organs. GAG accumulation progressively leads to cell dysfunction and death causing impairment of most organ/systems, including the brain in about two-thirds of the cases.

Incidence of MPS varies for each disorder and in different populations and ethnic groups, with overall prevalence going from 1.2 up to 16.9 over 100,000 live births recorded in the USA and Saudi Arabia, respectively [32].

MPS diagnosis normally proceeds from clinical suspicion, going through biochemical analysis, including urinary GAG and enzymatic assays, and is confirmed by molecular diagnosis [21].

Beyond symptomatic therapies, in the last 10–15 years treatment of these disorders has been mainly accomplished by enzyme supplementation, the so-called enzyme replacement therapy (ERT), available for MPS I, MPS II, MPS IVA, and MPS VI. Also, hematopoietic stem cell transplantation (HSCT) has been successfully applied almost exclusively to MPS I, while still debated for other MPS [9]. In addition to commonly requiring weekly hospitalization, ERT is an expensive procedure implicating important investments from the National Health Care Systems. Therefore, ethical and economic reasons impose its application to patients with a definite diagnosis.

In this study, we collected and evaluated the molecular diagnosis of a group of MPS patients enrolled in a multicenter Italian study, underlining its importance, together with enzymatic assays, to confirm the MPS clinical suspect and to reach a correct diagnosis. Importantly, a definite molecular diagnosis represents an essential tool for effective genetic counseling.

## Materials and methods

Seventy-one subjects affected by MPS were enrolled in a multicenter Italian study, financed by the Italian Ministry of Education, University and Research (MIUR), planning, among others, the collection of clinical, biochemical, and molecular data during a follow-up evaluation. All procedures performed were in accordance with the ethical standards of the institutional research committee and with the 1964 Helsinki declaration and its later amendments. Informed consent was obtained from all individual participants included in the study. Here, we report data related to genetic diagnoses, which were performed in different laboratories applying, in most cases, standard molecular methods (PCR amplification and Sanger sequencing); in few specific cases, second- or third-level analyses were conducted aiming to detect gross deletions, rearrangements, CNVs, or deep intronic variants. Genetic data was extrapolated from a web-based platform shared among the different units. For the patients with no molecular diagnosis available, molecular genetic analysis was not feasible at the time of enrollment in the present study, as patients’ DNA samples were not available. Variants reported in the original diagnostic reports were checked using Name Checker (https://www.mutalyzer.nl/name-checker) and, when necessary, were re-annotated on the basis of the most recent HGVS nomenclature (version 15.11; http://www.hgvs.org/nutnomen/). In addition, the novel missense variants were in silico tested for pathogenicity with four different prediction tools: DANN [47], MutationTaster [52], GERP [16], SIFT [55]; moreover, a structural evaluation of the impact of the amino acid substitution on the enzyme structure was performed through the tool HOPE [65]. Finally, all variants were further analyzed, (re-)classified according to the criteria suggested by the American College of Medical Genetics and Genomics (ACMG) [50] and submitted to ClinVar database (https://www.ncbi.nlm.nih.gov/clinvar/) where they will be publicly available.

## Results and discussion

In the population examined, 16 patients were affected by MPS II (22.5%), 13 by MPS IIIA (18.3%), 12 by MPS IVA (16.9%), 9 by MPS I (12.7%), 9 by MPS IIIB (12.7%), 7 by MPS VI (9.9%), 2 by MPS IIIC, 2 by MPS IVB, and 1 patient was affected by MPS IIID (Table 1). Molecular diagnosis had been performed for 61 subjects out of 71 enrolled in the study (85.9%). Age at diagnosis varied for the different disorders and severity. Generally, severe forms were diagnosed earlier, likely due to the early appearance of first clinical signs and symptoms. This is evident when different forms are described within the same disease, as in MPS I and MPS II. A significant difference between severe and attenuated forms was registered in our population for MPS I, with an average of 1.1 and 6.3 years at diagnosis respectively. This was also registered for MPS II, with an average of 2.9 and 6.6 years at diagnosis respectively (this last value was calculated excluding patient P14, a subject presenting a mild phenotype, who was diagnosed at 44.3 years of age).

**Table 1:** Genotypes of the patients enrolled in the study

Disease	Patient code (MIM reference number)	Clinical form	Age at diagnosis	Nucleotide change	Predicted amino acid change	Zigosity	Enzymatic activity
P1	MPSI (MIM # 607016, 607015, 607014)	Mild	4.9	c.793G>C	p.(Gly265Arg)	HT	0 nmol/mg/h (n.r. 13.1–23.5)
				c.1205G>A	p.(Trp402*)		
P2		Mild	4.5	NA	–	–	0 nmol/mg/h (n.r. 13.1–23.5)
P3		Severe	1.5	c.1487C>G	p.(Pro496Arg)	HT	0 nmol/mg/h (n.r. 13.1–23.5)
				c.1727+1G>A	–		
P4		Mild	4	NA	–	–	0.008 nmol/mg/h (n.r. 13.1–23.5)
P5^(a)^		Mild	0.3^(b)^	c.1205G>A	p.(Trp402*)	HT	0 nmol/mg/h (n.r. 20–180)
				c.1603C>T	p.(Leu535Phe)		
P6^(a)^		Mild	4.6	c.1205G>A	p.(Trp402*)	HT	0 nmol/mg/h (n.r. 20–180)
				c.1603C>T	p.(Leu535Phe)		
P7		Severe	1.5	NA	–	–	0 nmol/mg/h (n.r. 3.3–59)
P8		Mild	13.7	NA	*–*	–	NA
P9		Severe	0.3	c.979G>C	p.(Ala327Pro)	HT	NA
				c.1045G>T	p.(Asp349Tyr)		
P10	MPSII (MIM # 309900)	Mild	15.1	c.1264T>C	p.(Cys422Arg)	HE	NA
P11		Mild	3.7	NA	–	–	23.2 nmol/ml/4 h (n.v. 448; 802)
P12		Mild	5.3	c.187A>G	p.(Asn63Asp)	HE	*NA*
P13		Severe	2.7	c.359C>G	p.(Pro120Arg)	HE	0.5 nmol/mg/4 h (n.r. 2.1–6)
P14		Mild	44.3^(c)^	c.1563A>T	p.(Glu521Asp)	HE	0 nmol/mg/4 h (n.r. 18–57)
P15		Severe	7.8	c.811A>T	p.(Arg271Trp)	HE	0.2 nmol/mg/4 h (n.r. 18–57)
P16		Severe	1.7	c.589_592del	p.(Pro197Thrfs*15)	HE	1.2 nmol/mg/h (n.v. > 36)
P17		Severe	4.7	c.1478G>C	p.(Arg493Pro)	HE	0 nmol/mg/4 h (n.r. 18–57)
P18		Severe	1.8	NA	–	–	0 nmol/mg/h (n.r. not available**)**
P19		Severe	1.1	del ex 1–7 (2 deletions in tandem with 2 duplications 1.2 Mb distally to IDS gene)	–	HE	0.5 nmol/mg/h (n.v. 69.2)
P20		Severe	2.7	c.1403G>A	p.(Arg468Gln)	HE	0.3 nmol/mg/h (n.v. 32)
P21		Mild	2.4	c.708G>A	p.(Lys236Lys)	HE	0.8 nmol/mg/h (n.v. 31)
P22		Severe	4	c.592G>A	p.(Asp198Asn)	HE	NA
P23		Severe	3	Homologous recombination IDS-IDS2	–	HE	0.24 nmol/mg/4 h) (n.r. 35–80)
P24		Severe	1	c.1400C>T	p.(Pro467Leu)	HE	0.66 nmol/mg/4 h (n.r. 13.2–58.2)
P25		Severe	1	deletion of the whole IDS gene	–	HE	0 nmol/mg/4 h (n.r. 13.2–58.2)
P26	MPSIIIA (MIM # 252900)	Severe	6	c.220C>T	p.(Arg74Cys)	HO	0 nmol/mg/17 h (n.r. 1.8–5.8)
P27		Severe	NA	c.197C>G	p.(Ser66Trp)	HO	0.5 nmol/mg/17 h (n.r. not a vailable)
P28		Severe	4.4	c.220C>T	p.(Arg74Cys)	HT	0.6 nmol/mg/17 h (n.r. 15.3–41.3)
				c.364G>A	p.(Gly122Arg)		
P29		Severe	3	c.448C>T	p.(Arg150Trp)	HT	2.6 nmol/mg/17 h (n.v. 27)
				c.1147del	p.(His383Thrfs*30)		
P30		Severe	7.8	c.734G>A	p.(Arg245His)	HT	NP
				c.1339G>A	p.(Glu447Lys]		
P31		Severe	3.6	c.118T>A	p.(Tyr40Asn)	HT	0.8 nmol/mg/17 h (n.r. 4.3–5.6)
				c. 197C>T	p.(Ser66Trp)		
P32		Mild	18^(c)^	c.617G>C	p.(Arg206Pro)	HO	0.5 nmol/mg/17 h (n.r. 2.9–9.4)
P33		Severe	5.3	c.544C>T	p.(Arg182Cys)	HO	0.18 nmol/mg/17 h (n.r. 3–6)
P34^(a)^		Severe	6	c.1080del	p.(Val361Serfs*52)	HO	NA
P35^(a)^		Severe	2	c.1080del	p.(Val361Serfs*52)	HO	NA
P36		Severe	4	c. 197C>T	p.(Ser66Trp)	HT	NA
				c.220C>T	p.(Arg74Cys)		
P37		NA		c.221G>A	p.(Arg74His)	HT	NA
				c.542A>G	p.(His181Arg)		
				c.1097del	p.(Ser366Thrfs*47)		
P38		Severe	1.7	NA	–	–	0.03 nmol/mg/17 h (n.r. 4.1–12)
P39	MPSIIIB (MIM # 252920)	Severe	14.4	NA	–	–	0.001 nmol/mg/h (n.v. 0.1)
P40		Severe	3.5	NA	–	–	0 nmol/mg/h (n.r. not available**)**
P41		Severe	8.1	NA	–	–	0.006 OD (c.v. > 100)
P42		Severe	6	c.230T>G	p.(Val77Gly)	HT	0.88 nmol/mg/h (n.r. 2.7–4.9)
				c.1241A>G	p.(His414Arg)		
P43		Severe	3	c.419A>G	p.(Tyr140Cys)	HT	0 nmol/mg/h (n.r. 2.7–4.9)
				c.1144G>T	p.(Asp382Tyr)		
P44^(a)^		Mild	4	c.874G>A	p.(Gly292Arg)	HT	NA
				c.1928G>A	p.(Arg643His)		
P45^(a)^		Mild	3	c.874G>A	p.(Gly292Arg)	HT	NA
				c.1928G>A	p.(Arg643His)		
P46		Severe	1.5	c.874G>A	p.(Gly292Arg)	HO	NA
P47		Severe	5	c.274T>C	p.(Tyr92His)	HO	NA
P48^(a)^	MPSIIIC (MIM # 252930)	Mild to severe	9	c.852-1G>A	–	HO	NA
P49^(a)^		Mild to severe	6	c.852-1G>A	–	HO	0 nmol/mg/h (n.r. not available**)**
P50	MPS IIID (MIM # 252940)	Severe	5.5	c.814C>T	p.(Gln272*)	HO	0.12 nmol/mg/17 h (n.r. 26.5–35.5)
P51	MPSIVA (MIM # 253000)	NA	4.4	c.1A>G	(p.Met1?)	HT	1.7 nmol/mg/17 h (n.r. 17–53)
				c.1156C>T	p.(Arg386Cys)		
P52		Mild	4	c.346G>A	p.(Gly116Ser)	HT	0 nmol/mg/17 h (n.r. 12–19)
				NC_000016.9: g.88836836_88899132del62296	–		
P53^(a)^		Severe	1	c.1520G>T	p.(Cys507Phe)	HO	8.1 nmol/mg/17 h (n.r. 74.7–116.7)
P54^(a)^		Severe	1.5	c.1520G>T	p.(Cys507Phe)	HO	0.6 nmol/mg/17h (n.r 12-19)
P55		NA	2.8	c.29G>A	p.(Trp10*)	HO	0.6 nmol/mg/17 h (n.r. 19–42.7)
P56		Severe	1.9	c.29G>A	p.(Trp10*)	HO	0.36 nmol/mg/h (n.r. 3.7–18.6)
P57		Very mild	8	c.463G>A	p.(Gly155Arg)	HT	0.2 nmol/mg/17 h (n.r. 40–170)
				c.1002 + 307G>C	–		
P58		NA	2.7	c.1043C>A	p.(Thr348Asn)	HO	0.5 nmol/mg/17 h (n.r. 9–15)
P59		NA	2.6	c.1219A>C	p.(Asn407His)	HT	NA
				c.1507_1508del	p.(Lys503Valfs*226)		
P60		Severe	5.2	c.1519T>C	p.(Cys507Arg)	HO	0.6 nmol/mg/h (n.r. 4.4–19)
P61		Very severe	1.9	c.347G>T	p.(Gly116Val)	HT	0 nmol/mg/h (n.r. 3.7–18.6)
				c.868G>A	p.(Gly290Ser)		
P62		Moderate	2.8	c.29G>A	p.(Trp10*)	HT	0.14 nmol/mg/h (n.v. > 20.8)
				c.1519T>C	p.(Cys507Arg)		
P63	MPS IVB (MIM # 253010)	Mild	7.4	Single nucleotide substitution	Missense variant	HT	12.1 nmol/mg/17 h (n.r. 90–250)
				c.817_818delinsCT	p.(Trp273Leu)		
P64		Mild	12.7	c.817_818delinsCT	p.(Trp273Leu)	HT	14.4 nmol/mg/h (n.v. 237)
				c.1480-2A>G	–		
P65	MPS VI (MIM # 253200)	Severe	2	c.323G>T	p.(Gly108Val)	HO	48 nmol/mg/h (n.r. 95.8–162.8)
P66		Mild	3	c.725A>C	p.(His242Pro)	HT	0 nmol/mg/h (n.r. 95.8–162.8)
				c.1213+6T>C	–		
P67		Severe	1	c.944G>A	p.(Arg315Gln)	HO	0 nmol/mg/h (n.r. 95.8–162.8)
P68		Severe	1.8	c.1213+6T>C	–	HO	0 nmol/mg/h (n.r. 134–302)
P69		Severe	1.6	c.(898+1_899-1)_(1142+1_1143-1)del	–	HO	17.2 nmol/mg/h (n.r. 84.2–218.3)
P70		Severe	1	c.(898+1_899-1)_(1142+1_1143-1)del	–	HO	0 nmol/mg/h (n.r. 84.2–218.3)
P71		Mild to moderate	2.4	c.245T>C	p.(Leu82Pro)	HO	0.13 nmol/mg/h (n.r. 0.72–3.75)

*HT*, heterozygous; *HO*, homozygous; *HE*, hemizygous; *NA*, not available: *NP*, not performed for enzymatic substrate unavailability; *n.r*., normal range; *n.v*., normal value in healthy subject; *OD*, optical density

^(a)^P5 and P6, P34 and P35, P44 and P45, P48 and P49, and P53 and P54 are couple of siblings

^(b)^P5 was not included in the calculation of the mean age at diagnosis since the patient was monitored from birth due to the affected sibling

^(c)^The age at diagnosis for patients P14 and P32 was considered as outlier data and excluded from the calculation of the mean value

As for MPS IIIA and MPS IIIB, in our population diagnosis was reached on average around 5 years of age, except in one case of MPS IIIB, reported with an attenuated phenotype, and diagnosed at 18 years of age [23]. Most, if not all, MPS IVA cases were reported as severe and diagnosis was achieved on average at 3.2 years of age. The only 2 cases registered for MPS IVB are both reported with a mild phenotype and were diagnosed around 10 years of age. MPS VI, although commonly not affecting neurological functions, usually presents a severe skeletal phenotype, thus early diagnosis can be obtained. In our population, mean age at diagnosis for MPS VI was 1.8 years.

**Fig. 1:**
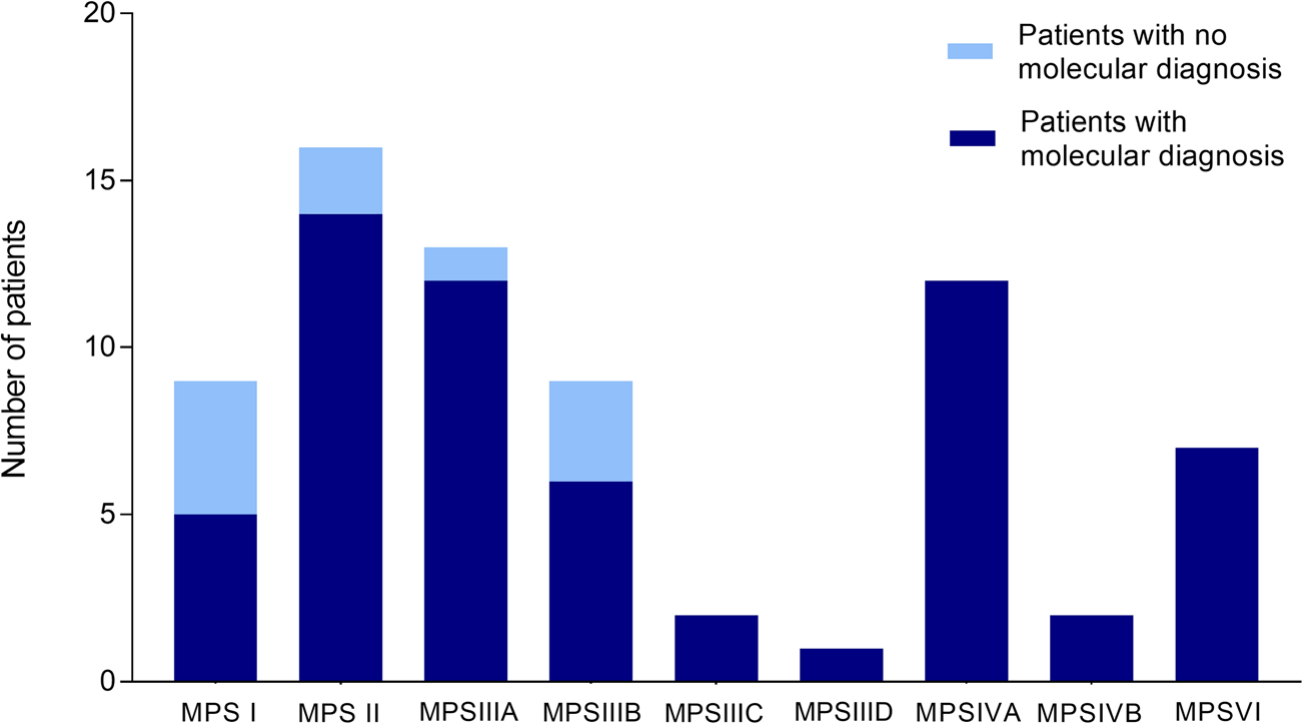
Number of patients with and without a molecular diagnosis

Distribution of diagnosed patients in the different MPS types is shown in Fig. 1. A missing molecular diagnosis was registered in 4/9 MPS I patients, 2/16 patients affected by MPS II, 1/13 MPS IIIA patients, and 3/9 MPS IIIB patients. Thus, for MPS I and MPS II patients, for whom either HSCT or ERT have been available for several years, a total of 6 out of 25 patients did not receive a molecular definition. Three of them underwent HSCT and 3 underwent ERT, based on clinical and biochemical evaluations. For 4 of them, molecular analysis of the genes was not feasible at the time of diagnosis. For all of them, it was never completed afterward.

As for the MPS IIIA and B patients, 4 of which remained molecularly undiagnosed, we suggest this may be due to the delay with which MPS III patients are sometimes clinically recognized and also to the lack of treatments for all MPSIII, which may cause a limited clinical follow-up with time.

Overall, of the 10 patients with no molecular diagnosis, 7 had been clinically diagnosed 20 or more years ago, while most of the patients (70%) with molecular diagnosis came to the clinical observation more recently in the last 15 years. Thus, we could argue that in the past, molecular diagnosis likely did not receive significant attention in the completion of the patient diagnosis, given also the fact that some MPS genes were identified in the early 2000s. Moreover, an evaluation of the timing elapsed between clinical/enzymatic diagnosis and molecular diagnosis showed in general, for all MPS taken together, a delay of about 4.6 years of the molecular diagnosis with respect to the clinical/enzymatic diagnosis, with a range from 0 up to 22 years.

A summary of all identified genotypes is reported in Table 1. Since for some patients, variants had been identified several years ago, we checked all of them and when necessary we re-annotated them according to the most recent HGVS nomenclature. On the whole, we report 87 variants, of which 67 are unique. Nine of the reported variants had never been described in the literature before.

Analysis of the genetic alterations identified in the examined population showed that, as expected, most of the variants were missense (about 70%), followed by small deletions (9%), large deletions/rearrangements (7.5%), splicing (7.5%), nonsense (4.5%) and sense variants (1.5%) (Table 2). In the context of MPS, we need to consider that while small variants may be identified in all disorders, other variants, as complex rearrangements, may be more easily encountered in subjects affected by MPS II due to homologous recombinational events between the iduronate 2-sulfatase gene (*IDS*) and its pseudogene (*IDS2*) [48].

**Table 2:** Variants identified in the patients enrolled in the study and their predicted ACMG classification

Gene (reference sequences)	Nucleotide change	Predicted amino acid change	Accession number	Reference	Predicted ACMG classification
*IDUA* (NM_000203.4; NP_000194.2)	c.793G>C	p.(Gly265Arg)	rs369090960	[73]	Likely pathogenic
	c.979G>C	p.(Ala327Pro)	rs199801029; ClinVar ID: 167190	[10]	Likely pathogenic
	c.1045G>T	p.(Asp349Tyr)	–	[66]	Likely pathogenic
	c.1205G>A	p.(Trp402*)	rs121965019; ClinVar ID: 11908	[53]	Pathogenic
	c.1487C>G	p.(Pro496Arg)	rs772416503; ClinVar ID: 496861	[4]	Likely pathogenic
	c.1603C>T	p.(Leu535Phe)	–	[24]	Not enough evidence
	c.1727+1G>A	–	–	[7]	Likely pathogenic
*IDS* (NM_000202.7; NP_000193.1)	c.187A>G	p.(Asn63Asp)	–	[26]	Not enough evidence
	c.359C>G	p.(Pro120Arg)	–	[27]	Likely pathogenic
	c.592G>A	p.(Asp198Asn)	ClinVar ID: 221210	[1]	Likely pathogenic
	c.589_592del	p.(Pro197Thrfs*15)	–	Novel	Pathogenic
	c.708G>A	p.(Lys236Lys)	–	[49]	Likely pathogenic
	c.811A>T	p.(Arg271Trp)	–	Novel	Likely pathogenic
	c.1264T>C	p.(Cys422Arg)	–	[38]	Not enough evidence
	c.1400C>T	p.(Pro467Leu)	–	[22]	Not enough evidence
	c.1403G>A	p.(Arg468Gln)	ClinVar ID:10498	[71]	Pathogenic
	c.1478G>C	p.(Arg493Pro)	–	[46]	Likely pathogenic
	c.1563A>T	p.(Glu521Asp)	–	Novel	Likely pathogenic
	Deletion of the whole IDS gene	–	–	[56]	Pathogenic
	Homologous recombination IDS-IDS2	–	–	[36]	Pathogenic
	Deletion ex 1–7 (2 deletions in tandem with 2 duplications 1.2 Mb distally to IDS gene)	–	–	[74]	Pathogenic
*SGSH* (NM_000199.4; NP_000190.1)	c.118T>A	p.(Tyr40Asn)	–	[17]	Likely pathogenic
	c.197C>G	p.(Ser66Trp)	rs104894637; ClinVar ID:5111	[8]	Pathogenic
	c.220C>T	p.(Arg74Cys)	rs104894636; ClinVar ID:5108	[69]	Likely pathogenic
	c.221G>A	p.(Arg74His)	ClinVar ID:550504	[11]	Likely pathogenic
	c.364G>A	p.(Gly122Arg)	rs761607612; ClinVar ID:518269	[11]	Likely pathogenic
	c.448C>T	p.(Arg150Trp)	–	[3]	Not enough evidence
	c.542A>G	p.(His181Arg)	–	Novel	Not enough evidence
	c.544C>T	p.(Arg182Cys)	rs529855742; ClinVar ID:523015	[17]	Likely pathogenic
	c.617G>C	p.(Arg206Pro)	ClinVar ID:5118	[37]	Likely pathogenic
	c.734G>A	p.(Arg245His)	rs104894635; ClinVar ID:5107	[8]	Pathogenic
	c.1080del ^(a)^	p.(Val361Serfs*52)	–	[69]	Pathogenic
	c.1097del	p.(Ser366Thrfs*47)	–	Novel	Likely pathogenic
	c.1147del	p.(His383Thrfs*30)	–	Novel	Likely pathogenic
	c.1339G>A	p.(Glu447Lys)	rs104894639; ClinVar ID:5114	[8]	Likely pathogenic
*NAGLU* (NM_000263.3; NP_000254.2)	c.230T>G	p.(Val77Gly)	–	[5]	Likely pathogenic
	c.274T>C	p.(Tyr92His)	–	[51]	Likely pathogenic
	c.419A>G	p.(Tyr140Cys)	–	[76]	Likely pathogenic
	c.874G>A	p.(Gly292Arg)	rs1358994052; ClinVar ID:553021	[13]	Not enough evidence
	c.1144G>T	p.(Asp382Tyr)	–	Novel	Not enough evidence
	c.1241A>G	p.(His414Arg)	rs768814260; ClinVar ID:552833	[70]	Likely pathogenic
	c.1928G>A	p.(Arg643His)	ClinVar ID:1563	[75]	Not enough evidence
*HGSNAT* (NM_152419.2; NP_689632.2*)*	c.852-1G>A	–	ClinVar ID: 556501	[20]	Pathogenic
*GNS* (NM_002076.3; NP_002067.1)	c.814C>T	p.(Gln272*)	–	[6]	Pathogenic
*GALNS* (NM_000512.4; NP_000503.1)	c.1A>G	(p.Met1?)	–	[60]	Likely pathogenic
	c.29G>A	p.(Trp10*)	–	[12]	Pathogenic
	c.346G>A	p.(Gly116Ser)	–	[61]	Likely pathogenic
	c.347G>T	p.(Gly116Val)	–	[40]	Likely pathogenic
	c.463G>A	p.(Gly155Arg)	rs398123438; ClinVar ID:93178	[12]	Likely pathogenic
	c.868G>A	p.(Gly290Ser)	–	[59]	Likely pathogenic
	c.1043C>A	p.(Thr348Asn)	–	[14]	Likely pathogenic
	c.1156C>T	p.(Arg386Cys)	rs118204437; ClinVar ID:700	[43]	Likely pathogenic
	c.1219A>C	p.(Asn407His)	rs749578474	[12]	Not enough evidence
	c.1507_1508del	p.(Lys503Valfs*226)	–	[14]	Likely pathogenic
	c.1519T>C	p.(Cys507Arg)	–	[14]	Likely pathogenic
	c.1520G>T	p.(Cys507Phe)	ClinVar ID:93169	[40]	Not enough evidence
	NC_000016.9: g.88836836_88899132del62296	–	–	[14]	Likely pathogenic
	c.1002+307G>C	–	–	[15]	Not enough evidence
*GLB1* (NM_000404.3; NP_000395.2)	Single nucleotide variation	Missense variant	–	Novel; Morrone A et al. in publication	–
	c.817_818delinsCT	p.(Trp273Leu)	–	[44]	Likely pathogenic
	c.1480-2A>G	–	rs587776526; ClinVar ID: 946	[39]	Pathogenic
*ARSB* (NM_000046.4; NP_000037.2)	c.245T>C	p.(Leu82Pro)	–	Novel	Not enough evidence
	c.323G>T	p.(Gly108Val)	rs768802200; ClinVar ID:559769	[28]	Not enough evidence
	c.725A>C	p.(His242Pro)	ClinVar ID:559808	[42]	Not enough evidence
	c.944G>A	p.(Arg315Gln)	rs727503809; ClinVar ID:166694	[68]	Likely pathogenic
	c.(898+1_899-1)_(1142+1_1143-1)del	–	ClinVar ID: 559663	[2]	Pathogenic
	c.1213+6T>C	–	ClinVar ID:559692	[42]	Pathogenic

^(a)^Variant c.1080del in SGSH gene was previously reported as c.1091delC, according to Scott et al. 1995 [54]

**Fig. 2:**
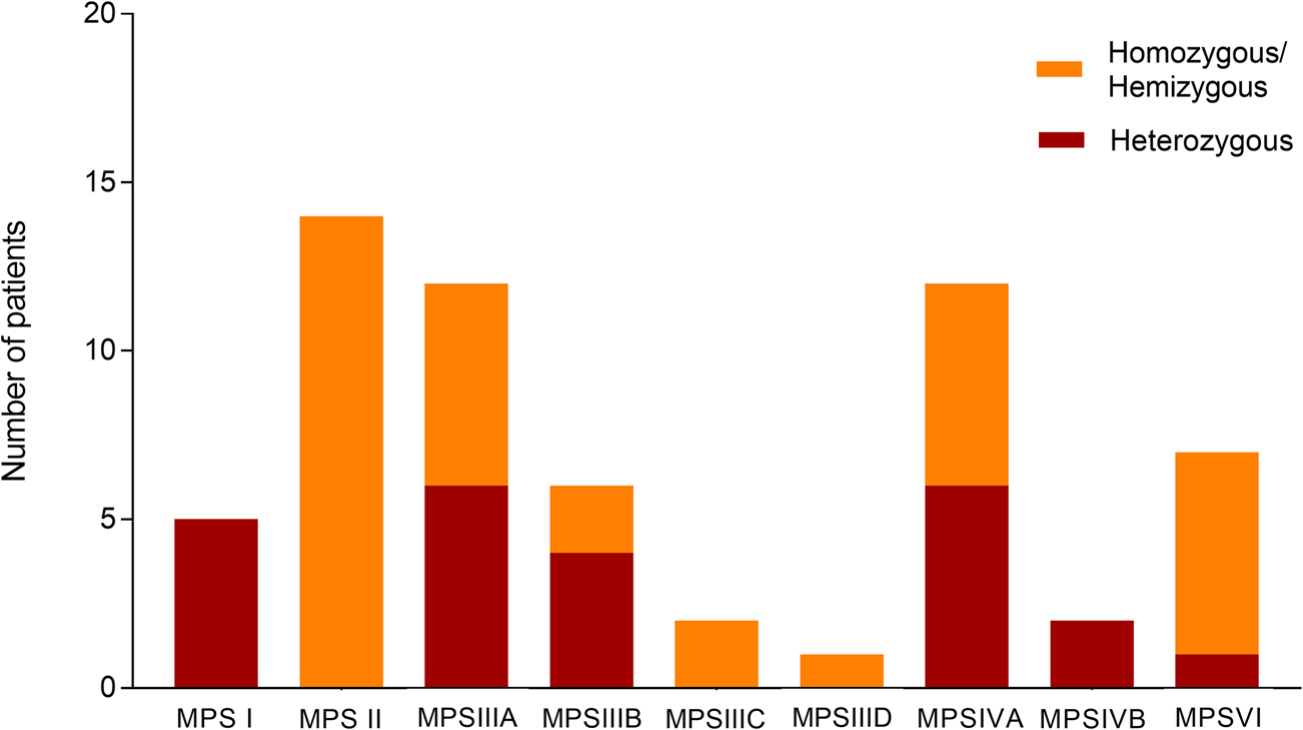
Number of heterozygous and homozygous/hemizygous patients in each MPS

Finally, Fig. 2 shows, for each MPS, the number of patients carrying the variant in hemizygosis or homozygosis. Homozygous mutations have been confirmed in parents for 15 out of 23 homozygous patients, thus excluding the presence of deletions on a single allele. In one case, P27, only the mother was analyzed, while for patient P32, only the sister was analyzed. For patient P69, carrying a homozygous deletion of exon 5 of ARSB gene, the homozygosis status was unequivocally confirmed by mRNA analysis. For the remaining 5 patients, analysis of any parents or relatives was not available. However, for 4 of them parents were consanguineous, rendering unlikely the chances of “apparent homozygosity.”

Homozygous patients provide a valuable tool to evaluate the in vivo effect of each specific variant, helping to possibly define genotype/phenotype correlations. In this respect, we underline as such evaluations may only be possible based on a strict collaboration between clinics, defining the patient phenotype based on shared severity criteria, and laboratory, carrying out the molecular analysis. In addition, information on parents’ ethnicity and consanguinity provided to the laboratory would be very important for the interpretation of the obtained molecular results and for genetic counseling.

Apart from MPS II which, being an X-linked disorder, is always caused by a single pathogenic variant (hemizygous condition), 6/12 of the MPS IIIA as well as 6/12 of the MPS IVA patients presented a condition of homozygosis. Also, 2 MPS IIIB, 2 MPS IIIC, and 1 MPS IIID patients showed a condition of homozygosis. Interestingly, 6 out of the 7 MPS VI patients presented a condition of homozygosity for different pathogenic variants; this reflects a general condition for this disorder in which a wide analysis of the literature has shown that more than 55% of the patients present genomic homozygosity for different pathogenic variants of the ARSB gene [58].

Eleven of the 23 homozygous patients had consanguineous parents, this explaining homozygosity, in these cases likely independent from the geographical distribution of the variants. It is known that gross deletions or gene rearrangements are commonly associated with severe clinical phenotypes. In our study, this was confirmed in 3 MPS II severe patients, whose causative mutations were represented by the deletion of several exons of the *IDS* gene (P19 in Table 1), deletion of the whole *IDS* gene (P25) and an event of homologous recombination between *IDS* gene and pseudogene (P23). This was also seen in MPS VI, where the deletion of exon 5 of the ARSB gene was associated with a severe phenotype (P69 and P70, Table 1). Table 3 summarizes the phenotypes found in this study in homozygous patients carrying point mutations and those described in the literature with the same variants.

**Table 3:** List of point mutations found in hemizygosis and homozygosis and the corresponding phenotypes found in our cohort of patients and in the literature

Gene	Nucleotide change	Predicted amino acid change	Phenotypes of homozygous/hemizygous patients described in our cohort	Phenotypes of homozygous/hemizygous patients described in literature
IDS	c.187A>G	p.(Asn63Asp)	Mild (P12)	Mild [26]; intermediate [30]; attenuated [62]; attenuated [18]
	c.359C>G	p.(Pro120Arg)	Severe (P13)	Severe to intermediate [27]; severe [33]
	c.592G>A	p.(Asp198Asn)	Severe (P22)	Severe [1]
	c.708G>A	p.(Lys236Lys)	Mild (P21)	Intermediate [49]
	c.1264 T>C	p.(Cys422Arg)	Mild (P10)	severe [38]
	c.1400C>T	p.(Pro467Leu)	Severe (P24)	Phenotype not reported [22]
	c.1403G>A	p.(Arg468Gln)	Severe (P20)	Severe [71]; severe [57]; severe [67]; four severe patients [63]; severe [34]; three severe patients [35]; severe [31]; severe [25]; severe [18]
	c.1478G>C	p.(Arg493Pro)	Severe (P17)	Phenotype not reported [46]
SGSH	c.197C>G	p.(Ser66Trp)	Severe (P27)	Two severe, three intermediate, one unknown [17]
	c.220C>T	p.(Arg74Cys)	Severe (P26)	Severe [41]; unknown [72]
	c.544C>T	p.(Arg182Cys)	Severe (P33)	No homozygotes described in literature
	c.617G>C	p.(Arg206Pro)	Mild (P32)	The same patient reported in the present study was des cribed in [23]
	c.1080del	p.(Val361Serfs*52)	Severe (P34, P35)	Severe [69]; three severe patients [37]; two severe patients [3]; severe [19]; severe [64]
NAGLU	c.274T>C	p.(Tyr92His)	Severe (P47)	No homozygotes described in literature
	c.874G>A	p.(Gly292Arg)	Severe (P46)	No homozygotes described in literature
HGSNAT	c.852-1G>A	–	Mild to severe (P48, P49)	The same patients reported in the present study were described in [20]
GNS	c.814C>T	p.(Gln272*)	Severe (P50)	The same patient reported in the present study was described in [6]
GALNS	c.29G>A	p.(Trp10*)	Severe (P56)	Unknown [12]; severe [60]
	c.1519T>C	p.(Cys507Arg)	Severe (P60)	Severe [14]
	c.1520G>T	p.(Cys507Phe)	Severe (P53, P54)	No homozygotes described in literature
ARSB	c.323G>T	p.(Gly108Val)	Severe (P65)	No homozygotes described in literature
	c.944G>A	p.(Arg315Gln)	Severe (P67)	Intermediate [68]; severe [45]; five patients with not reported phenotype [29]
	c.1213+6T>C	–	Severe (P68)	The same patient reported in the present study was described in [42]

As for the other 11 *IDS* variants identified in this study, mainly represented by missense mutations or small deletions, most of which is previously described, they were in the literature variably associated with either severe or attenuated or mild phenotypes (Table 3). Five of them are reported as associated with the same phenotype reported previously in other patients: 2 of them confirming a mild phenotype (P12 and P21 in Table 3) and 3 of them confirming a severe phenotype (P13, P20, and P22 in Table 3). However, the small number of patients analyzed for each variant does not allow drawing conclusions on these genotype-phenotype correlations.

With regards to homozygous patients identified in all MPS, but in MPS I and MPS IVB, a genotype-phenotype correlation analysis was conducted in 6 MPS IIIA patients, 3 of which confirming a previously described phenotype (P26, P34, and P35 in Table 3). For the other 3 homozygotes, no confirmation of previously described phenotypes was possible, thus not allowing to hypothesize any genotype-phenotype correlation. Concerning MPS IVA, 2 homozygous patients out of 6 confirmed previously described severe phenotypes (P56 and P60). Finally, as for MPS VI, none of the homozygotes’ phenotypes unequivocally correlated with previously described patients (Table 3), therefore we could not confirm any genotype-phenotype correlations. Of the 2 patients showing a mild phenotype, one was carrying in homozygosis the variant c.245T>C (P71 in Table 1), previously undescribed. Other genotype-phenotype correlations for this gene could be inferred indirectly; as for the splicing variant c.1213+6T>C, this was identified in homozygosis in a severe patient and in heterozygosis in a mild patient, compound heterozygote for the variant c.725A>C. This last variant, still presenting “not enough evidence” of pathogenicity according to ACMG classification, could confer the mild phenotype.

In addition, the novel missense variants [IDS: c.811A>T; IDS: c.1563A>T; IDS: c.542A>G, SGSH: c.542A>G, NAGLU: c.1144G>T, and ARSB: c.245T>C] were evaluated for pathogenicity with four different prediction tools (DANN, Mutation Taster, GERP and SIFT) which all confirmed the potential pathogenicity of the tested variants. This was also confirmed by the application of the tool HOPE that predicted potential remarkable structural changes in the enzyme structures which could affect the catalytic activity of the examined proteins. Results of the mentioned in silico evaluations are reported in the Online Resource.

Finally, all variants were re-evaluated according to the recent classification of ACMG (Table 2). The results of this evaluation evidenced that more than half of the variants (almost 56%) fell into the “Likely pathogenic” class, 31% into the “Not enough evidence” class, and the remaining resulted “Pathogenic” (13%).

## Conclusions

The present study underlines the need to complete the diagnostic workup of MPS patients previously diagnosed on a biochemical basis, through the identification and, possibly, the validation of the related gene variants. Molecular diagnosis is essential to confirm an enzyme deficit and provides diagnostic certainty to disorders for which the application of available treatments requires hospitalization and is extremely expensive. Moreover, we strongly recommend a molecular diagnosis based on the analysis of the “trio” instead of the sole proband, thus allowing the correct definition of the family inheritance and the identification of the de novo variants, which require different counseling. Finally, we suggest a periodical re-annotation of the variants according to the most recent version of HGVS nomenclature and solicit laboratories to perform their deposition in public databases (as LOVD, ClinVar, etc.), freely available to all clinicians and researchers. These recommendations will help obtain a complete and correct diagnosis of mucopolysaccharidosis, rendering also possible genetic counseling.

In these last years, molecular diagnosis of MPS, and in general of monogenic-inherited disorders, has taken advantage of new analytical approaches which have widen the possibility of investigation and have shortened the timing of diagnosis. This includes next-generation sequencing, applied to targeted genes (panels) or, more widely, to exome (whole exome sequencing, WES) or genome analyses (whole genome sequencing, WGS). Moreover, validation of the new genomic variants can now partly take advantage of the availability of several public databases collecting exomic or genomic data from large scale sequencing projects (ExAC, gnomAD, etc.).

These tools, used with the appropriate critical evaluation, may now allow more rapid and correct identification and validation of the genomic variants associated with a specific clinical phenotype.

## Electronic supplementary material

ESM 1 (DOCX 383 kb)

## Abbreviations

ACMG: American College of Medical Genetics and Genomics
CNV: Copy number variations
ERT: Enzyme replacement therapy
GAG: Glycosaminoglycans
HGVS: Human Genome Variation Society
HSCT: Hematopoietic stem cells transplantation
MPS: Mucopolysaccharidosis(es)
WES: Whole exome sequencing
WGS: Whole genome sequencing
