# Supplementary material for: Molecular diagnosis of patients affected by mucopolysaccharidosis: a multicenter study
Source: Eur J Pediatr. 2019 Feb 26;178(5):739–53. doi: 10.1007/s00431-019-03341-8 (PMC6459791; doi:10.1007/s00431-019-03341-8)
Supplement: Supplementary file 1 — (DOCX 383 kb) [file 431_2019_3341_MOESM1_ESM.docx]

**Online Resource**

**Molecular Diagnosis of Patients Affected by Mucopolysaccharidosis: a Multicentre Study**

*European Journal of Pediatrics*

Alessandra Zanetti^1,2^, Francesca D'Avanzo^1,2^, Laura Rigon^1,2^, Angelica Rampazzo^1^, Daniela Concolino^3^, Rita Barone^4^, Nicola Volpi^5^, Lucia Santoro^6^, Susanna Lualdi^7^, Francesca Bertola^8^, Maurizio Scarpa^1,2^ and Rosella Tomanin^1,2,*^

^1^ *Laboratorio di Diagnosi e Terapia delle Malattie Lisosomiali, Dept. of Women's and Children's Health, University of Padova, Padova, Italy*

^2^ *Fondazione Istituto di Ricerca Pediatrica Città della Speranza, Padova, Italy*

*^3^ Department of Pediatrics, University of Catanzaro, Catanzaro, Italy*

^4^*Department of Clinical and Experimental Medicine, Child Neurology and Psychiatry, University of Catania, Catania, Italy*

^5^ *Department of Life Sciences, University of Modena and Reggio Emilia, Modena, Italy*

^6^ *Department of Clinical Sciences, Division of Pediatrics, Polytechnic University of Marche, Ospedali Riuniti, Presidio Salesi, Ancona, Italy*

^7^ *Laboratorio di Genetica Medica e Biobanche* *, Istituto Giannina Gaslini, Genova, Italy*

*^8^School of Medicine and Surgery, University of Milano Bicocca, Monza, Italy*

*Corresponding author: Rosella Tomanin, e-mail: rosella.tomanin@unipd.it, tel. +390498211264, fax +390498217478

| **Gene** | **c.DNA annotation** | **protein annotation** | **DANN** | **Mutation Taster** | **GERP** | **SIFT** |
| --- | --- | --- | --- | --- | --- | --- |
| **IDS** | c.811A>T | p.(Arg271Trp) | 0,9986 | disease-causing | 5,37 | damaging |
| **IDS** | c.1563A>T | p.(Glu521Asp) | 0,9987 | disease-causing | 5,77 | damaging |
| **SGSH** | c.542A>G | p.(His181Arg) | 0,9976 | disease-causing | 4,26 | damaging |
| **NAGLU** | c.1144G>T | p.(Asp382Tyr) | 0,9943 | disease-causing | 4,54 | damaging |
| **ARSB** | c.245T>C | p.(Leu82Pro) | 0,9986 | disease-causing | 4,52 | damaging |

**Online Resource_Table I**: Results of the *in silico* analysis of pathogenicity of the novel missense variants, performed through the tools DANN (https://cbcl.ics.uci.edu/public_data/DANN/) [4], Mutation Taster (http://www.mutationtaster.org/)[5], GERP (http://mendel.stanford.edu/SidowLab/downloads/gerp/) [6] and SIFT (http://sift.bii.a-star.edu.sg/) [7]. The conservation score calculated by GERP ranges from -12.3 to 6.17 with 6.17 being the score for the highest conservation.

| **Gene** | **c.DNA annotation** | **protein annotation** | **Difference of the mutated amino acid compared with the wild type one** | | | **Bonds potentially affected by the amino acid substitution** |
| --- | --- | --- | --- | --- | --- | --- |
|  |  |  | **Dimension** | **Charge** | **Hydrophobicity** |  |
| **IDS** | c.811A>T | p.(Arg271Trp) | bigger | neutral  (wild type = negative) | more hydrophobic | hydrogen bond with Ser 282; salt bridge with Asp 478 |
| **IDS** | c.1563A>T | p.(Glu521Asp) | smaller | --- | --- | hydrogen bonds with Trp 502 and Tyr 523;  salt bridge with Arg 468 |
| **SGSH** | c.542A>G | p.(His181Arg) | bigger | positive  (wild type = neutral) | --- | interaction with metal ion calcium;  hydrogen bond with Arg 182 |
| **NAGLU** | c.1144G>T | p.(Asp382Tyr) | bigger | neutral  (wild type = negative) | more hydrophobic | hydrogen bonds with Val 390 and Tyr 391 |
| **ARSB** | c.245T>C | p.(Leu82Pro) | smaller | --- | --- | the mutated amino acid causes an empty space in the protein core |

**Online Resource Table II**: Results of the evaluation performed by HOPE [8] (http://www.cmbi.umcn.nl/hope/) of the potential structural effect of the mutated amino acid for each of the new missense variants reported in this manuscript.


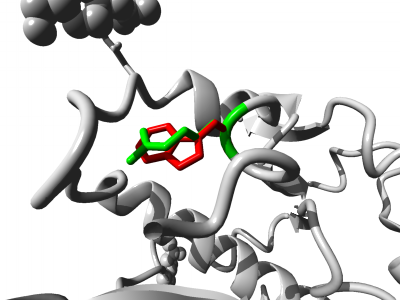

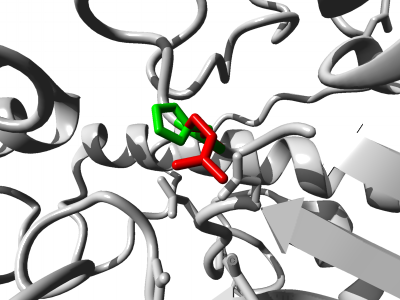

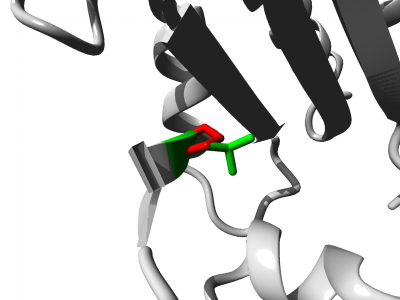


**ARSB: p.(Leu82Pro)**

**SGSH: p.(His181Arg)**

**IDS: p.(Glu521Asp)**

**IDS:p.(Arg271Trp)**

**rp)**


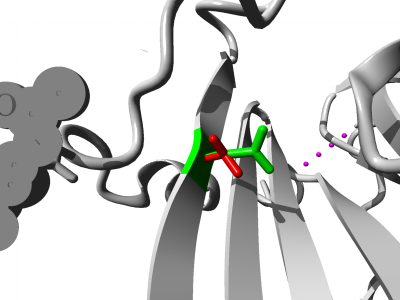

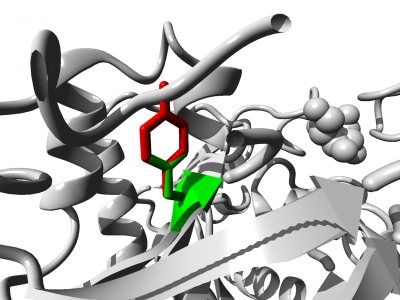


**NAGLU: p.(Asp382Tyr)**

**Online Resource_Figure I:** Close-up of the amino acid substitutions in the structural prediction performed by HOPE [8] (http://www.cmbi.umcn.nl/hope/) for each of the five novel missense variants reported in our study. The protein is grey-coloured and the side chain of wild-type and mutant amino acids are shown coloured green and red respectively.
